# Cortex: Unravelling the final frontier in pain

**DOI:** 10.1080/24740527.2017.1329298

**Published:** 2017-05-22

**Authors:** Rohini Kuner

**Affiliations:** Heidelberg University, Germany

Speaker Abstract: A timely and fundamental question in the neurosciences revolves around how functional specificity is generated in highly redundant brain circuits. Several regions are activated in the human brain during a pain percept, which have not been interrogated functionally so far. In this talk, I will briefly review some of the recently acquired insights and present new data from our laboratory addressing the function and specificity of medial prefrontal cortical circuits in pain.

We identified a novel functional role of an important subdivision of the cingulate cortex, namely the mid-cingulate cortex (MCC), in central plasticity mediating the transition from acute to chronic pain. In functional mapping studies, the MCC emerged as a key nodal point in cortical and subcortical circuits activated in pain. With a view towards this goal, we employed *in vivo* optogenetic manipulations, viral-based circuit tracing and functional analyses in mice. Taken together with previous seminal work on the rostral (pregenual) subdivision of the anterior cingulate (rACC), our work reveals a functional dichotomy between the rACC and the MCC in creating the multidimensional experience of pain.

Recent studies in humans emphasize the importance of oscillatory activity in the brain in creating and modulating the percept of pain. In this context, I will present data on the analysis of brain activity rhythms in rodents during acute and chronic pain and discuss their functional relevance to pain. Overall, our data suggest that multiple brain regions can trigger nociceptive hypersensitivity independently of peripheral nociceptor input into the brain. These findings can explain changes in pain sensitivity reported in patients in the absence of (or persisting following healing of) obvious injuries or physical pathologies. Moreover, they provide a mechanistic basis for exacerbation of pain by psychosocial factors, such as stress and anxiety, that may deregulate activity in cortical circuits.

